# Development of a program theory for osteoporosis patient education in Denmark: a qualitative study based on realist evaluation

**DOI:** 10.1186/s12877-024-04957-8

**Published:** 2024-04-16

**Authors:** Mette Rubæk, Marie Broholm-Jørgensen, Susan Andersen, Pernille Ravn Jakobsen, Mette Juel Rothmann, Bente Langdahl, Mette Friberg Hitz, Teresa Holmberg

**Affiliations:** 1 National Institute of Public Health, University of Southern Denmark, Copenhagen, Denmark; 2 National Research Center for Bone Health, Zealand University Hospital, Køge, Denmark; 3 Department of Public Health, Research Unit of General Practice, University of Southern Denmark, Odense, Denmark; 4 Steno Diabetes Center Odense, Odense University Hospital, Odense, Denmark; 5 Department of Endocrinology, Odense University Hospital, Odense, Denmark; 6 Department of Clinical Research, University of Southern Denmark, Odense, Denmark; 7 Department of Endocrinology, Aarhus University Hospital, Aarhus, Denmark; 8 Department of Clinical Medicine, Aarhus University, Aarhus, Denmark; 9 Centre for Childhood Health, Copenhagen, Denmark

**Keywords:** Osteoporosis, Patient education, Program theory, Mechanism of change

## Abstract

**Background:**

Osteoporosis patient education is offered in many countries worldwide. When evaluating complex interventions like these, it is important to understand how and why the intervention leads to effects. This study aimed to develop a program theory of osteoporosis patient education in Danish municipalities with a focus on examining the mechanisms of change i.e. what is about the programs that generate change.

**Methods:**

The program theory was developed in an iterative process. The initial draft was based on a previous published systematic review, and subsequently the draft was continually refined based on findings from observations (10 h during osteoporosis patient education) and interviews (individual interviews with six employees in municipalities and three health professionals at hospitals, as well as four focus group interviews with participants in patient education (in total 27 informants)). The transcribed interviews were analyzed using thematic analysis and with inspiration from realist evaluation the mechanisms as well as the contextual factors and outcomes were examined.

**Results:**

Based on this qualitative study we developed a program theory of osteoporosis patient education and identified four mechanisms: motivation, recognizability, reassurance, and peer reflection. For each mechanism we examined how contextual factors activated the mechanism as well as which outcomes were achieved. For instance, the participants’ motivation is activated when they meet in groups, and thereafter outcomes such as more physical activity may be achieved. Recognizability is activated by the participants’ course of disease, which may lead to better ergonomic habits. Reassurance may result in more physical activity, and this mechanism is activated in newly diagnosed participants without previous fractures. Peer reflection is activated when the participants meet in groups, and the outcome healthier diet may be achieved.

**Conclusions:**

We developed a program theory and examined how and why osteoporosis patient education is likely to be effective. Understanding these prerequisites is important for future implementation and evaluation of osteoporosis patient education.

**Supplementary Information:**

The online version contains supplementary material available at 10.1186/s12877-024-04957-8.

## Background

Osteoporosis is a common chronic bone disease with an estimated prevalence of 27.6 million people in Europe [1]. The prevalence increases markedly with age; among people aged 80 + the prevalence is 37% [1]. Osteoporosis has consequences for the individual, primarily due to the related risk of fractures. Globally, 158 million people were estimated to be at high fracture risk in 2010 and this number is likely to double by 2040 [2]. The occurrence of fractures may result in decreased quality of life [3, 4], pain [5, 6], institutionalization, and death [7]. Osteoporosis can be treated, and the related fracture risk may be prevented by initiating pharmacological treatment as well as improving diet, considering supplements, being physical active, and preventing falls [8–12].

When diagnosed with osteoporosis, the patient's self-image may change, as they can feel older and embarrassed, which can affect their social life [13]. Patients with osteoporosis may also experience dependency on others and shifting roles at home because they cannot maintain their daily activities [13]. Therefore, some patients find it helpful to discuss these problems and share experiences with other patients [14].

Patient education is often used by patients to gain support and be able to manage the consequences of osteoporosis. According to WHO, osteoporosis patient education should give the participants the opportunity to express their concerns and discuss their expectations with a health professional and gain support from other participants. Further, WHO recommends that osteoporosis patient education consist of training and information about the disease, available treatment, diet, exercise, lifestyle, and prevention of falls and fractures, [10] and thereby encourage bone healthy behaviors. A multifaceted and multidisciplinary approach is also recommended in other studies [15–18]. Osteoporosis patient education is carried out in many countries worldwide [19–21]. The content of the programs differs, but most programs include information about osteoporosis [15, 22–27], medication and diet [15, 22–29], prevention of falls and fractures [15, 22, 26, 28], as well as training and information about physical exercise [24, 26, 30, 31].

Osteoporosis patient education could be defined as a complex intervention, as it includes different components, it targets different behaviors, and it requires different skills from health professionals [32, 33]. Furthermore, complexity may arise when the intervention or program in this case interacts with the context at the patient education sessions or outside the sessions [32].

The effectiveness of osteoporosis patient education has been evaluated worldwide in previous studies [15, 24, 26–31], but because of inconsistent findings it is difficult to draw overall conclusions about its effect [21]. When evaluating complex interventions, the Medical Research Counsel (MRC) highlights the importance of developing a program theory that illustrates how the intervention could lead to effects and under what circumstances [34]. The program theory should be described clearly, and a visual presentation may be helpful [34].

We are not aware of any studies presenting a program theory of osteoporosis patient education. Moreover, prior studies have not shown how the effects of patient education are dependent on contextual factors and do not examine how and why the interventions work (i.e., they do not examine the mechanisms of change). In a previous published systematic review, we found that examination of mediators is absent in many effectiveness studies, and therefore we recommended that future studies should examine mediators, which could contribute to an understanding of the mechanisms of osteoporosis patient education [21]. Likewise, other researchers have highlighted the need for studies examining the mechanism of the interventions, rather than only examining the effects [35–38]. When examining the mechanisms, it is possible to find an explanation for the effectiveness of an intervention [39] and thereby open the ‘black box’ that describes how and why the intervention works [40]. Moreover, the mechanisms contribute with important information for future implementation and evaluation of the interventions.

A program theory and an examination of the mechanisms is essential for the development, for implementation, and evaluation of new osteoporosis patient education programs. Furthermore, as osteoporosis patient education in Danish municipalities has not been evaluated systematically, a program theory and an understanding of the mechanisms are important prerequisites for evaluation of these existing programs and the selection of relevant outcomes for the evaluation.

When examining mechanisms of change qualitative studies should preferably be conducted [35, 41]. This study aimed to develop a program theory of osteoporosis patient education in Danish municipalities with a focus on examining the mechanisms of change i.e. what is about the programs that generate change.

## Methods

The study was conducted as an explorative study, including observations and interviews and with realist evaluation [39] as theoretical inspiration. Moreover, it was based on knowledge from a systematic review undertaken by the research group, which has been published [21].

### Theoretical inspiration

Realist evaluation is a theory-driven approach, which examines how and why interventions work, as well as under what circumstances [39], hence aiming to describe the process by which the effects occur, rather than just describing whether effects occur. This is done by developing, testing and refining context-mechanism-outcome (CMO) configurations [39]. A CMO configuration can be defined as a part of a program theory by representing assumptions about how and why interventions work. In this study, we examined the contextual factors, the mechanisms of change, as well as the outcomes of osteoporosis patient education while describing important paths in the program theory, which is of special importance for future implementation and evaluation of osteoporosis patient education.

The development of our program theory was based on the elements put forward by Gertler et al. [42], meaning that the program theory outlined inputs, activities, outputs, and outcomes. Moreover, we identified the mechanisms of osteoporosis patient education using a definition within realist evaluation, in which a mechanism describes what it is about the programs that generate change (i.e., outcomes) [44]. In particular we are inspired by Dalkin and colleagues (2015) definition of mechanism that contains two components, namely the *resources* from the intervention and the derived *reasoning* in the individual [39, 43]. In this line of thought, the mechanisms are the response the intervention activities trigger from the individual participant. Of relevance for this study, the premise of this definition is, that it is the participants in the osteoporosis patient education that make it work (or not) depending on how they respond to the resources it offers to them [44]. Thus, a mechanism is a theory describing the association between an exposure and an outcome [39]. In realist evaluation the context activates the mechanisms, i.e., a particular contextual factor triggers a specific mechanism, which again results in an outcome [39]. Therefore, we examined not only the mechanisms, but also which contextual factors activate the mechanisms as well as which outcomes may be achieved.

### Study setting

In Denmark, osteoporosis patient education is offered at hospitals as well as in municipalities, although most osteoporosis patient education is carried out in municipalities that have the main responsibility for rehabilitation. Each municipality can decide whether they will implement osteoporosis patient education, and it is estimated that around 22 of 98 municipalities offer osteoporosis patient education [45]. The programs differ across municipalities, but typically the participants meet face-to-face in groups of approximately 10 persons, once a week for eight weeks. The programs most often include a combination of knowledge dissemination and physical exercises [46, 47]. The physical exercises vary across municipalities but often they focus on balance, strength training, and aerobic exercises. The referral differs as well, as some municipalities require a referral from General Practitioner (GP) or hospital, whereas others do not. For this study, we chose six municipalities, which were widely representative regarding the location (rural or urban as well as distribution across the country) and size of the municipality, the participants, the content, and referral to the program. Information about the municipalities offering osteoporosis patient education was retrieved from existing overviews [45, 46]. The programs in the six municipalities are described in Table 1.

**Table 1:** Overview of patient education in six municipalities

Municipality	Participants	Number of participants	Duration	Content	Teachers	Referral from GP	Informants in this study
Municipality 1	Persons with osteoporosis	Approx. 12	8 weeks, 2 times a week	Exercises: Balance, strength training, and aerobic exercises Lectures: Information about osteoporosis, diet, medication, physical exercise, ergonomics, etc	Physiotherapist, dietician, and visits from the pharmacy and the Danish Osteoporosis Society	Referral not needed	1 employee, 5 participants
Municipality 2	Persons with osteoporosis	Approx. 10	12 weeks, 2 times a week	Exercises: Balance, strength training, and aerobic exercises Lectures: Information about osteoporosis, diet, medication, ergonomics, pain management, etc	Physiotherapist and, if necessary, dietician	Referral from GP or hospital	1 employee, 5 participants
Municipality 3	Persons with osteoporosis	Approx. 10	9 weeks, once a week	Exercises: Balance, strength training, and aerobic exercises Lectures: Information about osteoporosis, diet, medication, ergonomics, pain management, etc	Physiotherapist, occupational therapist, dietician, nurse, and visit from the Danish Osteoporosis Society	Referral not needed	1 employee, 3 participants
Municipality 4	Persons with osteoporosis	Approx. 12	8 weeks, 2 times a week	Exercises: Balance, strength training, and aerobic exercises Lectures: Information about osteoporosis, diet, medication, ergonomics, pain management, habits, etc	Physiotherapist, occupational therapist, nurse, and dietician	Referral from GP or hospital	1 employee, 5 participants
Municipality 5	Both persons with osteopenia and osteoporosis	-	10 weeks, 2 times a week	Exercises: Primarily strength training Lectures: Information about osteoporosis, diet, medication, ergonomics, etc	Physiotherapist, occupational therapist, and dietician	Referral from GP or hospital	1 employee
Municipality 6	Persons with osteoporosis	6–10	6 weeks, once a week	Exercises: Primarily strength training Lectures: Information about osteoporosis, diet, medication, ergonomics, pain management, etc	Physiotherapist, occupational therapist, nurse, and dietician	Referral from GP or hospital	1 employee

The physical exercises vary across municipalities. Balance training includes e.g. exercises on the floor where the participants stand on one leg. Strength training includes e.g. exercises on machines where the participants do three sets with 10 repetitions. Aerobic exercises include e.g. dancing, walking/running, and jumping

### Procedure

We developed the program theory based on a qualitative study and a previously published systematic review of osteoporosis patient education [21]. During the qualitative study, different stakeholders were involved in exploring their shared understanding [34]. The decision about who should be involved and which methods should be used depends on the purpose of developing a program theory [48]. Our aim was to get an understanding of the elements in osteoporosis patient education to be used in future implementation and evaluation of the programs. We conducted observation and interviews, as it is advantageous to combine them if each method is used to elaborate the other [49]. In addition, a multimethod evidence base is recommended within realist evaluation [39].

In the first draft of the program theory, we included the outcomes identified in the previously published systematic review in which we examined the effectiveness of osteoporosis patient education [21]. Thereafter we continuously added the other elements of the program theory based on observations and interviews in the qualitative study. During this process, the draft was revised several times and carefully aligned with the findings. Lastly, a final version of the program theory was decided by researchers within the field of intervention and osteoporosis research. Consequently, the development of the program theory was an iterative process as described in realist evaluation [39, 50] and recommended for research in complex interventions [34], and therefore the development of the program theory followed an abductive argument.

Observation and interviews were conducted from May 2021 until December 2021. The research project has been approved by Research Ethics Committee, University of Southern Denmark (approval ID 20/70420). Informed consent was retrieved from participants in osteoporosis patient education (hereafter also called participants) after they were provided with written and oral information. All methods were performed in accordance with the Declaration of Helsinki.

### Observation

To get first-hand experience with the activities in osteoporosis patient education as well as the interactions between participants, the setting, and the atmosphere [51] i.e., the context, we carried out observations in four municipalities while sessions were held. Municipalities were eligible for observation if they provided osteoporosis patient education conducted in group settings and delivered face-to-face. A combination of observation and participant observation was conducted dependent on the content of the session. Observation implies no involvement from the researcher [51], and this was primarily carried out during educational sessions as participant observation would have been difficult without an osteoporosis diagnosis. Conversely, participant observation implies that the researcher takes part in an action [51], and this was carried out during physical exercises to avoid the participants feeling observed by the researcher. Observation and participant observation was carried out by the first author who has a background within public health and has experience with observations. Intermediate feedback was provided by several co-authors. No further observations were carried out when findings appeared across settings. 10 h of observations and participant observations was carried out (see Table 2).

**Table 2:** Overview of observation in municipalities

Municipality	Number of observed sessions	Content of sessions	Length of observation	Type of observation
Municipality 1	2	Combination of educational and exercise sessions	5 h (2 times 2.5 h)	Combination of observation and participant observation
Municipality 2	2	Exercise session	2 h (2 times 1 h)	Participant observation
Municipality 3	1	Combination of educational and exercise sessions	2 h	Combination of observation and participant observation
Municipality 4	1	Exercise session	1 h and 15 min	Participant observation

### Interviews

Interviews were conducted to gain insight into the informants’ knowledge, experiences, and understandings [51]. We conducted interviews with three groups of informants: participants in patient education, employees in municipalities (hereafter also called employees), and health professionals at hospitals (hereafter called health professionals). Interviews with participants provided insight into their experiences with the programs, and therefore participants who had completed most of a program were eligible for interviews. Interviews with employees in municipalities gave insight into the planning and execution of the programs, and therefore employees were eligible if they had been planning or executing programs. As some Danish hospitals also conduct osteoporosis patient education, interviews with health professionals gave insight into their knowledge about such programs. The health professionals were eligible for interviews if they had in-dept knowledge about a program at a hospital.

Interviews with health professionals and employees were conducted as individual interviews to explore their experiences with their specific programs [51]. Interviews with participants were conducted as focus group interviews to elicit reflection from them during interaction with other participants [51]. A total of 13 interviews were conducted: three interviews with health professionals, six interviews with employees, and four focus group interviews with participants, amounting to a total of 18 participants (three to five participants in each group). The average time per interview was 53 min.

Interviews with health professionals and employees took place online, whereas interviews with participants took place at facilities provided by the municipalities and were familiar to the participants. All interviews were performed by the first author who has experience with interviewing in health care settings. Several co-authors with similar experiences provided feedback between interviews. No further interviews were conducted when saturation was obtained, i.e. findings were mentioned by more informants. All interviews were recorded and afterwards transcribed verbatim (approximately 179 pages).

### Characteristics of informants

The characteristics of the 18 informants (participants in osteoporosis patient education) are shown in Table 3. Most of the participants were women (88%). Their ages ranged from 50 to 84 years (mean age 71 years). Most of the participants had completed the full program (61%), while others had yet to complete the last sessions. Their year of diagnosis ranged from 1995 until present, though most of them (56%) were diagnosed within the past two years. Eight participants had experienced vertebral fractures, and three had experienced other osteoporotic fractures. Most of them were retired and had an upper secondary or higher level of education.

**Table 3:** Overview of interviews with participants in osteoporosis patient education

Participant	Sex	Age group	Time since participation in patient education	Time of osteoporosis diagnosis	Vertebral (V) or other (O) fractures	Lives with others
Par1	M	70–74	Just finalized	2015–19	No	Yes
Par2	F	65–69	Just finalized	2020–24	No	No
Par3	F	75–79	Just finalized	2020–24	Yes (V)	Yes
Par4	F	75–79	Participated for 2 or 3 years ago	2015–19	Yes (O)	Yes
Par5	F	70–74	Participated for 2 years ago	2015–19	Yes (V + O)	Yes
Par6	M	60–64	Still participating	2020–24	No	Yes
Par7	F	80–84	Still participating, has few sessions left Has also participated 3 years ago	2005–09	Yes (V)	Yes
Par8	F	65–69	Still participating, has few sessions left	2015–19	Yes (O)	Yes
Par9	F	75–79	Still participating, has few sessions left	2010–14	Yes (V)	Yes
Par10	F	70–74	Still participating, has few sessions left	2020–24	No	Yes
Par11	F	50–54	Still participating, has one session left	2020–24	No	Yes
Par12	F	65–69	Still participating, has one session left	2020–24	Yes (V)	No
Par13	F	75–79	Still participating, has one session left	2020–24	Yes (V)	No
Par14	F	75–79	Participated for 3 or 4 years ago	2005–09	Yes (V)	Yes
Par15	F	60–64	Participated for 6 or 7 months ago	2020–24	Yes (V)	No
Par16	F	65–69	Participated for 6 or 7 months ago	1995–99	No	Yes
Par17	F	70–74	Participated for 6 or 7 months ago	2020–24	No	No
Par18	F	70–74	Participated for 6 or 7 months ago	2020–24	No	Yes

*M* Male, *F* Female

Among the six employees in municipalities, five were physiotherapists, and one was a nurse. Five of them were currently teaching on an osteoporosis patient education program. Most of them had participated in both the development and implementation of the programs.

The three health professionals at hospitals were nurses and had experience with osteoporosis patient education: two of them were currently teaching on the programs, and one was a researcher in the field of osteoporosis.

### Observation and interview guides

During observations unstructured field notes containing descriptions of the setting, content of the session, and the interaction between participants were collected.

For the interviews three different but related interview guides were developed. With inspiration from literature on program theory development [48], we developed the interview guides to contain questions regarding: barriers for osteoporosis patients, what the participants may achieve during patient education, why they achieve it, who needs to be involved, etc. The themes were related to the elements of the program theory, e.g., the effects of patient education, the mechanisms, and the contextual factors. In all interviews the interviewer began with an introduction of herself, the project, and the publication of results and afterwards the informants introduced themselves. In the focus group interviews, the interviewer asked questions which were then answered by informants, or the informants asked each other questions in a natural dialog. Furthermore, the participants were asked to write down the effects of patient education on Post-it notes, which were then discussed. During interviews with health professionals and employees the first draft of the program theory was presented to and commented on by the informants.

### Analysis

Due to our theoretical inspiration in realist evaluation, the analysis focused on the mechanisms, contextual factors, and outcomes. The interviews were analyzed using thematic analysis [52], which contains six steps: 1) Get familiar with data, 2) Generate initial codes, 3) Search for themes, 4) Review themes, 5) Define and name themes, 6) Report findings. Therefore, we initially listened to the recordings and read the transcriptions (step 1), whereafter we generated codes, e.g., “others” and “geography and practical matters” (step 2). Then, we found candidate themes, e.g., “surroundings” and “distance and duration” (step 3). Thereafter we read data within one theme as well as the entire data set across themes. In this process, some data were moved to another theme, some additional data were coded, and some themes were renamed (step 4). In the final steps, we described the essence of each theme (step 5) and wrote the results section (step 6). Examples from steps 2 to 5 are provided in Table 4. During the analysis, the field notes from observation were also examined to ensure that the themes were adequate and comprehensive. As the themes from the analysis emerged, relevant theories were identified and included to support our understanding and interpretation of the themes. Therefore, the application of theories comprised an additional analytical step (step 7).

**Table 4:** Examples from step 2 to 5 of the analysis of interviews

Step 2: Generate initial codes	Step 3: Search for themes	Step 4: Review themes	Step 5: Define and name themes
Others Geography and practical matters Try out Repetitions Body-consciousness Fear Exchange of experience Cohesion Patient stories Ergonomics Tacit knowledge New habits	**Theme: Surroundings** Codes: Others	**Theme: Family and friends** Codes: Others	**Theme: Training community** Essence: Training community is a contextual factor covering family, friends, or citizens the participants do physical exercises with
	**Theme: Distance and duration** Codes: Geography and practical matters	**Theme: Transport** Codes: Geography and practical matters	**Theme: Transport** Essence: Transport is a contextual factor covering the distance from the participants’ homes to the location where patient education is conducted
	**Theme: Try out** Codes: Try out, Repetitions, Body-consciousness	**Theme: Try out** Codes: Try out, Body-consciousness	**Theme: Recognizability** Essence: Recognizability is a mechanism describing how the participants recognize the physical feeling of, e.g., exercises after they have practiced those exact exercises
	**Theme: Fear** Codes: Fear	**Theme: Fear and reassurance** Codes: Fear	**Theme: Reassurance** Essence: Reassurance is a mechanism that describes how the participants are reassured during patient education. They are reassured because they may be afraid of, e.g., fracturing their bones
	**Theme: Interaction** Codes: Cohesion, Patient stories, Exchange of experience	**Theme: Peer reflection** Codes: Patient stories, Exchange of experience	**Theme: Peer reflection** Essence: Peer reflection is a mechanism describing how the participants can see themselves in one another, e.g., when they exchange experiences
	**Theme: New habits** Codes: Ergonomics, Tacit knowledge, New habits	**Theme: New ergonomic habits** Codes: Ergonomics, Tacit knowledge, New habits	**Theme: Ergonomic habits** Essence: Ergonomic habits is an outcome covering the participants learning how to move ergonomically, e.g., regarding cleaning, cooking, and gardening

The coding of data was both theory-driven and data-driven [52] as we coded the interviews into the overall elements of the program theory, i.e., inputs, activities, outputs, outcomes, context, and mechanisms. Furthermore, we added codes while reading the interviews without considering these overall elements to explore which other codes might appear. This was in accordance with our abductive approach.

The analysis was conducted in NVivo. The first author took the lead on step 1–3 with ongoing discussion with co-authors with experiences in qualitative research that collaboratively identified and developed themes within the data. This process also involved discussing emergent themes, examining supporting evidence from the data, and refining the themes (step 4–5). Moreover, the findings were also discussed at a workshop with all co-authors, who are researchers within intervention and osteoporosis research, and they were discussed with a team of qualitative researchers at the University of Southern Denmark (step 4–5). First author summarized and reported findings (step 6), which all co-authors commented on.

Below, the results of the analysis are described. For each mechanism, we examined which contextual factors activate the mechanism as well as which outcomes appear. This is in accordance with our theoretical inspiration, and moreover this description aims to illustrate the paths in the program theory.

## Results

From our analysis and in accordance with our research question, we identified the elements of a program theory of osteoporosis patient education with special attention to examining the mechanisms.

### Program theory

**Fig. 1:**
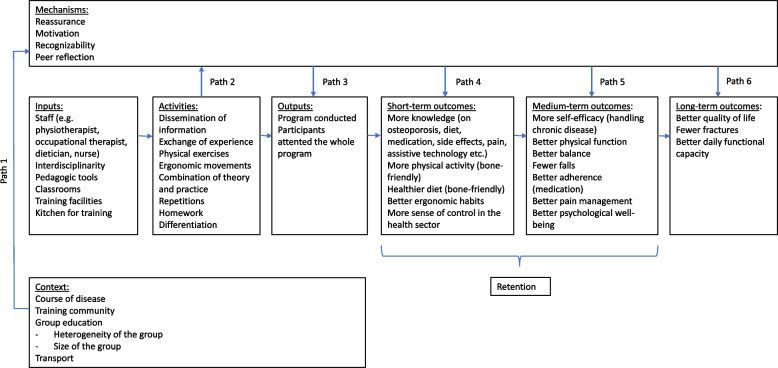
Program theory developed

Our program theory for osteoporosis patient education is presented in Fig. 1. Each element is described in Additional file 1, together with examples of empirical data. The inputs, activities, outputs, outcomes, and contextual factors are briefly described below, and the mechanisms are examined in detail in the following.

The inputs in the program theory include the means, which are necessary to carry out the program [42]. From our analysis, we found that staff is needed but different staff should also be able to cooperate across professional competences (interdisciplinarity). For instance, in one municipality it was observed that two physiotherapists and one nurse held a session with physical exercises, and afterwards they met to talk about the session and ensure interdisciplinarity. Pedagogic tools are also needed, as well as proper facilities (classrooms, training facilities, and perhaps a kitchen for training of ergonomic habits).

The activities consist of the work performed in the program [42]. Our analysis show how osteoporosis patient education should include dissemination of information, for instance, one employee in a municipality described which information they provide:

Emp5: […] so we get into, well, what is it you, I mean, what is osteoporosis and how does it come about, and how many people get it? […] And what lies, what are the recommendations about it? And, well, about diet, but also about ergonomics, and about physical exercises.

Furthermore, we found that the activities should include time for exchanging experiences. The participants should also do physical exercises as well as ergonomic movements during the sessions with a combination of theory and practice. The information and the exercises should be repeated, and there should be homework between each session. Finally, the teachers should be able to adapt the exercises to the participants’ functional capacity (differentiation).

The inputs and activities may result in outputs, which are the goods and services the program produces [42]. For osteoporosis patient education, we found that the outputs include the fact that the program is conducted and that the participants attend the whole program. In the six municipalities, the programs are conducted during 6–12 weeks with attendance 1–2 times a week, and from our analysis it seemed to be necessary to achieve the intended outcomes. One employee in a municipality described why it is necessary that they meet several times:

Emp1: […] But I would say that, you know, we’ve been together 16 times […] So that’s a long time to be able to carry out a process, instead of that you just get told things. I mean, if all of this, if it was boiled down to just two theme days, for example, I don’t think you would get the same out of it, because people, they just need to go home and work with it.

As a result of these outputs, several outcomes may be achieved. They are divided into short-term, medium-term, and long-term outcomes (presented in Fig. 1). For instance, one employee in a municipality described one of the overall goals of osteoporosis patient education:

Emp3: The long-term aim is hopefully to prevent fractures. You could say that the citizens who come here to the group, they’ve already had this diagnosis, so it’s not to prevent them getting osteoporosis, but in the long term to prevent fractures.

From our analysis, several contextual factors influence whether the outcomes are achieved, for instance, the participants’ course of disease (such as fractures, pain, or functional impairments). Furthermore, meeting in groups is influential including the size and the heterogeneity of the group. For instance, it was observed that some participants differ regarding age and functional abilities. Training communities in the participants’ local areas are important for doing physical exercises, and, finally, transportation is important for attending the patient education sessions.

### Mechanisms

Four mechanisms of osteoporosis patient education were identified: motivation, recognizability, reassurance, and peer reflection. In accordance with our theoretical inspiration from realist evaluation, we will describe the resources and reasoning of the mechanisms (Path 2) as well as which outputs and outcomes might occur (Paths 3–6). Finally, we will examine the contextual factors that activate each mechanism (Path 1).

### Motivation

Motivation was mentioned by the majority of informants. Motivation is initiated when the participants receive information and do homework (the resources of the intervention), whereby the participants are encouraged to engage in bone healthy activities (the reasoning in the individual).

Motivation was related to the performance of bone healthy activities, for instance, physical exercises and ergonomic habits. Motivation was interpreted with the Information-Motivation-Behavioral Skills Model (IBM) as described by Fisher & Fisher [53], in which motivation consists of personal motivation (attitudes toward personal performance of health promotion behaviors) and social motivation (social support for enactment of health promotion behaviors) [53, 54]. Therefore, motivation to engage in bone healthy activities consists of the participants’ attitudes towards these activities and the outcomes they would achieve, as well as their perceived support from others and willingness to comply with this.

Two activities were of importance for the participants’ motivation for initiating bone healthy activities: homework and dissemination of information. Several informants mentioned the importance of homework (Path 2), for instance, in one focus group where the participants talked about the homework consisting of physical exercises they were given for each session:

Par16: Well, I find it very difficult to motivate myself to do things on my own at home, that now you’ve got to get yourself together and do the exercises. But because you’ve signed up for this program, and you’re told that, well, there’s this homework, so suddenly it has become homework. Then you have to do it. When you’ve signed up for something, you have to do the things that come with it.

In this case, the homework motivated the participants to do physical exercises at home, thereby the outcome of more physical activity (Path 4) may be achieved. As well as doing homework, it is also important that the participants receive information on bone healthy activities during the program (Path 2). For instance, a health professional at a hospital explained that the participants need information in order to become motivated to engage in bone healthy activities:

Hea3: […] And then there’s still the concrete information about osteoporosis medicine and all that, which I actually think, if we didn’t include it, they would really miss it because they need to have some kind of motivation to do all the other things [bone healthy activities].

The participants also mentioned that the information provided motivated them to engage in bone healthy activities. Therefore, information and motivation were linked, which is in accordance with the IMB model. In the IMB model, information and motivation primarily work through behavioral skills to affect behavioral change. Behavioral skills includes both the patients’ objective abilities and their self-efficacy related to the particular behavioral change [54]. During osteoporosis patient education, the participants may achieve both of these skills, as they might obtain abilities, e.g., to be physically active, as well as self-efficacy related to physical activity. According to the Information-Motivation-Behavioral Skills Model (IBM model), these behavioral skills result in behavioral change, i.e., more physical activity (Path 4), as shown above.

### Contextual factors activating motivation

Contextual factors which were described to activate motivation were group education, the heterogeneity of the group, and the course of the disease. In the majority of interviews with participants, it was mentioned that meeting in groups (Path 1) increases their motivation to engage in bone healthy activities. In a focus group, one participant mentioned that being in a group motivated her to turn up and do physical exercises:

Par7: I think it’s really good to be in a group. Then you’re told how you should do it, and you get going and do it properly. I mean, I try to do it at home as well, but it’s not the same.

In this case, the participant’s motivation caused her to turn up for the sessions (Path 3) and do physical exercises.

On the other hand, the heterogeneity of the group (Path 1) can decrease motivation if one participant is quite different from the other participants. For instance, a health professional mentioned that if one participant smokes and has an unhealthy lifestyle compared to the others, then this participant may not be motivated because she feels that she is left out of the group.

Moreover, the contextual factor related to the course of the disease, for instance, the pain some participants experience (Path 1), was described to be of importance for motivating the participants. Several participants mentioned that their back pain motivates them to protect their back when they clean the house. This was discussed by two participants in a focus group:

Par13: when you say that you’ve figured out to take breaks, is it because you are in so much pain that you take breaks?

Par12: it’s to avoid pain.

In this case, the participants were motivated to take breaks when cleaning the house. They explained that they have learnt during patient education that they should take a break even before they feel pain. For these participants, the breaks are a good ergonomic habit, and therefore the outcome of better ergonomic habits (Path 4) may be achieved.

Besides showing how meeting in groups, the heterogeneity of the group, and the course of the disease can activate motivation to engage in bone healthy activities, we have also shown how motivation is initiated when the participants receive information and do homework. When the participants are motivated, outcomes such as more physical activity and better ergonomic habits may be achieved.

### Recognizability

Recognizability is a mechanism that appeared in many interviews. It is initiated, for example, when the participants do physical exercises and ergonomic movements (the resources of the intervention), and thereafter they find it easier to remember and perform these actions (the reasoning in the individual).

Recognizability means that the participants can recall what they have learnt on the program because they have learnt it with their bodies. This is comparable with *embodied knowledge* as defined by Merleau-Ponty [54]. Embodied knowledge means that “the body knows how to act”, and therefore one of the important features “is that the body, not the mind, is the knowing subject” [55]. The participants in osteoporosis patient education learn how to do the physical exercises and ergonomic movements, and this knowledge can become embodied knowledge. In a focus group, one participant described how the ergonomic movements in relation to laundry and cleaning had become embodied knowledge:

Par4: There are a lot of things [ergonomic movements], well, I just think it’s kind of second nature. There are a lot of things like that.

Activities that initiate this recognizability are physical exercises, ergonomic movements, a combination of theory and practice, repetitions, and homework (Path 2). Several informants mentioned that the participants do physical exercises, thereby becoming familiar with the physical feeling in the body. One employee in a municipality described how the participants learn to recognize the feeling of doing strength training. He described that many participants are unfamiliar with strength training, but during patient education they become familiar with the feeling of using their muscles and getting tired. This was also observed during the sessions, where some participants seemed to be unfamiliar with strength training in particular. Besides practicing physical exercises, the participants also practice ergonomic movements (e.g., related to gardening, cleaning, and cooking).

Embodied knowledge “can be better presented by performance than by verbal explanation” [55], and therefore it is useful that physical exercises and ergonomic movements are demonstrated and practiced. However, many informants mentioned that the participants should not only do physical exercises but they should also have a combination of theory and practice and they should do repetitions. One employee in a municipality described how she ensures this in her sessions:

Emp1: […] So it’s very much about getting the repetitions in. […] But because we keep talking about it, and we keep on with, well, quite simply, trying things out. So maybe that gives them an idea. Because, you know, we go out for a walk, and we see how it feels. Now we know where the muscle is, we can feel where it is ourselves, have I found it, haven’t I found it. Well, so, what happens when I walk slowly, oh, so then it disappears. What happens when I walk quickly, oh yes, then it perks up.

Homework between the sessions was mentioned by few informants. The homework gives the participant an opportunity to ask questions at the upcoming session. One employee in a municipality described the importance of homework:

Emp6: […] We have more success when we get them to go home and try during the program. […] Because then we see that when they do it during the program, there’s better potential for development afterwards. So, they don’t have to go home and reinvent the wheel when we’ve let them go, because then they won’t be able to ask the questions about it, and all that, which means that they actually succeed in getting started with some of it.

When the participants do the mentioned activities, the mechanism recognizability is initiated, and thereafter we have seen that outcomes such as more physical activity and better ergonomic habits (Path 4) may be achieved. Furthermore, several employees and health professionals described how recognizability is important for the overall outcome retention, i.e., that the participants continue doing the bone healthy activities. They explained that to do the bone healthy activities in the long run it is important to do, for example, homework, as mentioned by the employee above (Emp6).

### Contextual factors activating recognizability

Training communities (Path 1) were of importance for participants to implement the bone healthy activities. Because the participants do physical exercises together with a group of people during the sessions, recognizability can also be related to the feeling of practicing with other people (during the program). Therefore, recognizability can also be activated in the long run if the participants have training communities to do physical exercises with (after the program has ended). Some participants described how they need other people to do physical exercises with; otherwise, they would not do physical exercises:

Par12: I mean, I go to the gym because training alone – well, that’s probably not going to happen. So, I need all that with being with other people and…

The contextual factor related to the course of the disease (Path 1) can also activate recognizability. Few participants mentioned that during the sessions they discovered that they performed worse in physical exercises than they thought they would. The course of the disease may have left marks on their physical health, which they had not discovered. When they do physical exercises, they realize how their body feels now, and they learn to recognize this feeling. Thereby, they acquire new (or updated) embodied knowledge regarding physical exercises.

Therefore, contextual factors related to the course of the disease and training communities can activate the mechanism recognizability. As we have shown, five activities, namely physical exercises, ergonomic movements, a combination of theory and practice, repetitions, and homework, initiate the mechanism. As a result, outcomes such as more physical activity, better ergonomic habits, and retention may be achieved.

### Reassurance

Reassurance was a consistent theme in the vast majority of the interviews. Reassurance is initiated when the participants receive information and do physical exercises (the resources of the intervention), whereby the participants become calmer (the reasoning in the individual).

Reassurance means that the participants become calm after feeling fear. Reassurance was interpreted with elements of the Extended Parallel Process Model (EPPM) formulated by Witte [56], using which we examined the process whereby the participants move from being afraid to being calm.

Several employees and health professionals mentioned that osteoporosis patients may experience fear when they are diagnosed, which was also expressed by few participants. They worried about fracturing their bones, and this fear may itself result in physical inactivity. However, the participants can be reassured by receiving information and doing physical exercises (Path 2). One employee in a municipality mentioned an example of a participant who was on sick leave from work:

Emp3: […] But she was nervous, and she was scared of moving her body in general… But via that we could give her… We could help her feel safe to move during the session, we could give her knowledge about what is it you’re suffering from, what’s happening in the body when you have this osteoporosis. Well, then we got her ready to be able to start going to work again…

In this and many other examples, the participants were reassured that they can be physically active without being afraid of fractures. During observations, the participants also seemed to be calm when they performed aerobic exercises, even if this included running, jumping, etc.

The EPPM describes how a person experiencing fear should receive *efficacy information* containing two components: 1. *response efficacy*, which is information about the effectiveness of a recommendation and 2. *self-efficacy information*, which includes arguments that the person is able to follow the recommendation. This information should lead to a *danger control process* in which the person initiates the recommended activity [57]. As we have seen above, the participants in osteoporosis patient education receive this efficacy information, for instance, when they are informed about why it is important to be physically active (response efficacy) and when they participate in physical exercises and experience that they are able to do it (self-efficacy information). Thereby, the participants are reassured, and the outcome physical activity (Path 4) may be achieved.

Even though most participants are reassured, there is also a risk that their fear increases. One employee in a municipality mentioned that they had included sessions about pain and assistive technology, but these were dropped because they experienced that the participants became more afraid when hearing about how their life could turn out.

Although this fear should be avoided, experiencing fear can lead to the participants trying to prevent having more complications, as mentioned by one employee in a municipality:

Emp6: Well, sometimes at the start of our program we see that, actually some of the participants with less pain, that they get scared by hearing stories from the others. And of course, that’s where you have to turn it around and say: it’s okay to be nervous, it’s okay, because you should know that it is a risk, but you should also, to avoid getting to that point, then we should tackle these things, and these, and these.

Even though it is not the intention to evoke fear in the participants, the fear that does appear can be useful to achieve behavior change, which is also described in the EPPM model, in which fear is evoked more intentionally [58].

### Contextual factors activating reassurance

The course of the disease is a contextual factor of importance for activating reassurance (Path 1). Several employees and health professionals mentioned that it is especially the newly diagnosed as well as the participants without previous fractures who experience fear and therefore are reassured during patient education.

We highlighted above how some activities unintentionally may increase fear. One contextual factor can trigger the fear as well, namely the heterogeneity of the group (Path 1). Many employees and health professionals mentioned that the newly diagnosed may be afraid to hear stories from participants with more complications. Therefore, the participants at hospitals are divided into groups reflecting whether they have had vertebral fractures or not. One health professional described why it is important to separate them:

Hea3: […] And so we make groups with vertebral fractures and without vertebral fractures, because it can be kind of scary to be surrounded by participants that have had vertebral fractures and who have lots of stories about, so I sneezed and my vertebra collapsed. You know, these horror stories that participants who haven’t had a vertebral fracture don’t necessarily benefit from, because that doesn’t need to be their story. At the same time, we also have to include those who have had a vertebral fracture, and that this is their story, so they get acknowledgement, that this is happening for you, and how can we then help you in all of this?

Therefore, it was described as advantageous to ensure some homogeneity of the groups to minimize the risk of evoking fear.

We have shown how the mechanism reassurance is initiated when the participants receive information and when they do physical exercises, i.e., they receive *efficacy information* as described by Witte [57]. Thereby, they may achieve outcomes such as more physical activity. Reassurance is primarily activated in participants who are newly diagnosed and without previous fractures, whereas unintended fear can be activated in heterogeneous groups.

### Peer reflection

Peer reflection is a mechanism that is mainly initiated when the participants exchange experiences (the resources of the intervention) and thereby relate to one another (the reasoning in the individual).

Peer reflection means that the participants see themselves in one another and learn from each other. This is in accordance with *peer support* as defined by Mead et al. [58], which “is a system of giving and receiving help founded on key principles of respect, shared responsibility and mutual agreement of what is helpful” [58]. Peer reflection is initiated when the participants exchange experiences (Path 2), which was mentioned by almost all informants.

For instance, one employee in a municipality gave an example of a participant who was traveling across the country by train. She was in doubt if she could lift her suitcase into the train and was about to cancel the trip. However, when experiences were exchanged in the group, she was given advice on how to handle the problem with the suitcase, for instance ordering assistance from the staff. She could see herself and her situation in other participants’ similar situations. In the end, she went on the trip because of the advice she was given.

Moreover, exchange of experience was observed during more sessions as well as during almost all interviews where the participants asked each other questions related to bone healthy activities. For instance, during a focus group interview the participants discussed the importance of vitamin K and helped each other find out if they should take this supplement:

Par17: yes, but some of the calcium pills you get with [name on calcium pills], they have it with both D3 and K2, don’t they? And some places they write that it’s fine, and other places, well, we get the vitamin K we need through a normal diet? […].

Par15: but is vitamin K broken down, or does it build up [in the body]?

Par17: there’s K1 and K2.

Par14: it can definitely build up.

Par17: ah, but you can get very confused about that, if you ask me.

In this case, the participants exchanged experiences regarding vitamin K; they gave and received help from each other as described by Mead et al. [58]. During the interview, the participants indicated that they pay a great deal of attention to their diet, and therefore they could see themselves in one another and take examples from each other. They can help each other align their supplements to their diet, and therefore the outcome healthier diet (Path 4) may be achieved.

Exchange of experience is an important activity, but one health professional mentioned that the health professionals to some degree can imitate and replace this activity if they include patient stories in their teaching. However, one important advantage of exchange of experience between the participants themselves is that the participants can challenge each other in a way that health professionals cannot. Mead et al. [58] find that “as trust in the relationship builds, both people are able to respectfully challenge each other when they find themselves in conflict”. The aforementioned health professional also found it an advantage that the participants can challenge each other:

Hea1: […] What is also important in exchange of experience is that you as patient to patient, you can perhaps challenge each other in a different way than you sometimes think you can as a health professional. I mean, for example, say ‘so, are you going to go home and take some osteoporosis medicine after everything you’ve heard now?’ to someone who has never done it, because she doesn’t think she would do it.

Therefore, exchange of experience between participants allows them to challenge each other, which may be important for activating the mechanism of peer reflection.

### Contextual factors activating peer reflection

Two contextual factors were of importance, namely the size of the group and the heterogeneity of the group. As mentioned above, the heterogeneity of the group (Path 1) can have a negative influence if the participants become afraid when hearing the other participants’ stories. Conversely, the heterogeneity can also have a positive influence, as the participants get advice from those who are at another stage in the disease. For instance, during a focus group interview the participants agreed that even though they are at different stages, they all have challenges which the others benefit from hearing about.

When meeting in groups, the size of the group (Path 1) may be of importance for peer reflection, which was mentioned by many informants. One employee in a municipality explained how a group of 10 may interact better than a group of four:

Emp3: […] You could say, when we have 10 participants in a group […] it often gives a bigger, how shall I put it, a bigger kind of flow in the group in relation to the contact they seek out in each other, conversation, ping pong between the participants.

We have shown how the mechanism peer reflection is influenced by the size of the group as well as the heterogeneity of the group, which can have both positive and negative influence. The mechanism is initiated when the participants exchange experiences, thereby the outcome healthier diet may be achieved.

## Discussion

In this study, we developed a program theory of osteoporosis patient education, with a focus on examining the mechanisms of change. This theory describes what it is about the programs that lead change (i.e., outcomes). Four mechanisms explained how and why the programs work: motivation, recognizability, reassurance, and peer reflection. We have shown how the mechanisms are evoked by activities such as dissemination of information, exchange of experience, physical exercises, ergonomic movements, a combination of theory and practice, repetitions, and homework. A number of contextual factors activated the mechanisms: factors related to group education including the size and the heterogeneity of the group, the course of the disease, and training communities. Subsequently, more physical activity, better ergonomic habits, healthier diet, and retention may be achieved.

In accordance with our study, Jensen et al. (2016) identified factors, which are associated with implementing a bone healthy lifestyle after attending patient education [59]. One factor includes that the participants experience a need and motivation to implement the activities. Motivation may be evoked if the participants experience pain, which is similar to our findings. Another factor includes that the participants incorporate a bone healthy lifestyle into social activities. Likewise, we found that training communities play an important role for behavior change and doing physical exercises in the long run to achieve the medium and long-term outcomes.

We found that the participants may experience fear when diagnosed with osteoporosis but that they are reassured during patient education. This could be compared to Weston et al., who found that women diagnosed with osteoporosis were reassured regarding their treatment during consultations with their GP [60]. However, in our study we found that the participants are reassured because they receive information and do physical exercises, whereas Weston et al. found that trust in the GP was important for reassurance.

Furthermore, we found that the participants in some cases become more afraid during patient education sessions. Similarly, Nielsen et al., found that patients handle knowledge either by retaining hope or producing fear [61]. Nielsen et al. additionally found that patients participating in patient education in which experiences are exchanged become more confident and secure regarding their everyday life. Likewise, we found that exchange of experience is an important activity, which may result in undertaking more bone healthy activities.

### Implication of findings

This study contributes with insights and explanations about how and why osteoporosis patient education can work.

Our findings show what activities in osteoporosis patient education and what contextual factors activate the mechanisms of change and thereby potentially causing the intended outcomes *short-term* (more knowledge, more physical activity, healthier diet, better ergonomic habits, more sense of control in the health sector), *medium-term* (more self-efficacy, better physical function, better balance, better adherence to medication, better pain management, better psychological well-being) *and long-term* (better quality of life, fewer fractures, better daily functional capacity). Therefore, to implement osteoporosis patient education in practice one should consider these activities and contextual factors. We have shown that activities such as exchange of experience and a combination of theory and practice should be implemented, and contextual factors related to the course of the disease and meeting in groups should be considered.

Furthermore, we examined four mechanisms, that may result in intended outcomes. However, the mechanism motivation may be a prerequisite for achievement of more short-term outcomes, namely more physical activity, healthier diet, and better ergonomic habits, as one can ask whether, e.g., more physical activity can be achieved without motivation. Therefore, motivation probably should be activated in cases when patient education aims to improve physical activity, diet, and ergonomic habits, and maybe it should be activated together with other mechanisms. Future research should explore the specific role of motivation and the possible combination with other mechanisms in order to enable behavior change on the longer run.

In our program theory, we have illustrated how participation in osteoporosis patient education may result in several short-term, medium-term, and long-term outcomes. Nevertheless, most examples of achieved outcomes were related to short-term outcomes (Path 4), probably due to the design of the study. Most of the participants had just finished the patient education and therefore they could only provide examples of the short-term outcomes. Moreover, in cases where the municipalities had not made evaluations of the effectiveness of the programs, they could not provide evidence, but only assumptions, of the medium-term and long-term outcomes. To gain evidence, epidemiological studies are needed, which we will conduct at a later stage. In our program theory, we did include medium-term and long-term outcomes based on a systematic review [21], data from observations and interviews, as well as expert knowledge from researchers in intervention and osteoporosis research as described.

From the examples of short-term outcomes, we saw a focus on physical activity and ergonomic habits, which may reflect the activities in the programs as well as the competences of the teachers. The programs allocate a great deal of time to physical activity and ergonomic movements, whereas medication takes up less time. Physical activity and ergonomic movements are important for prevention of falls, management of pain and quality of life [26, 30, 62–64], however, adherence to medication is the most important intervention for prevention of fractures [8]. Therefore, the municipalities may benefit from even more interdisciplinarity and involvement, such as from physicians who could conduct sessions about medication. However, many employees described that most participants adhere to their medication, and therefore adherence may not be a problem for most of the participants.

To achieve long-term outcomes, participants' behavioral changes are crucial, meaning that participants should maintain bone-healthy activities, such as physical activity, healthier diets, and better ergonomic habits, over an extended period [8]. These behaviors require ongoing commitment and reinforcement to become integrated into individuals' daily routines and should endure over time. Therefore, consistent behavioral changes are vital for realizing the full potential of osteoporosis patient education in obtaining medium- and long-term outcomes (e.g., fewer fractures). However, it is important to note, that the recommendations for these bone-healthy activities depend on the course of the disease [65]. Therefore, the recommendations may change over the life course, and therefore it may be difficult for the teachers to disseminate information that the participants can use in the longer term, potentially decreasing the likelihood of the behavior change on the long run. This is a general limitation related to patient education conducted once in life. Maybe, the program should be repeated and adjusted several times during the life course or at least if the disease progresses [66]. This may apply for patient education but also for lifestyle interventions in general.

Furthermore, we have described how the groups in osteoporosis patient education in some respects seem to be heterogeneous, for instance, regarding age and functional abilities, but in other aspects they seem homogeneous, as they primarily consist of socioeconomically advantaged citizens. This is another limitation related to the programs, as socioeconomically disadvantaged citizens are not necessarily recruited. It may be because participation to some degree requires that the citizens are aware of the program and contact the municipality [67, 68]. Therefore, strategies to recruit socioeconomically disadvantaged citizens should be developed so that citizens from all social classes participate in osteoporosis patient education. Moreover, future research should examine to which extent our findings also apply to socioeconomically disadvantaged citizens.

Most of the mechanisms of change are initiated by activities which require a large number of resources. For instance, the mechanism recognizability is initiated when the participants do physical exercises, ergonomic movements, repetitions, homework, and when they have a combination of theory and practice. All of this requires resources such as staff, pedagogic tools, classrooms, and training facilities, and in addition it requires that the participants meet several times. Therefore, our findings indicate that osteoporosis patient education may require some resources and should be conducted over several sessions, and consequently one session would not be sufficient.

Finally, we found some examples of the participants experiencing fear. As described, this fear may be useful for motivating the participants, but evoking fear is not the intention of the patient education programs. At hospitals, the participants are divided into groups reflecting whether they have had vertebral fractures, and this may reduce the risk of evoking fear. Therefore, it may be beneficial to divide the participants in the municipalities in the same way, although this probably would not be a realistic setup as it requires many participants. However, the municipalities could make groups across municipalities, and to minimize transportation online participation or via other technology could be considered. Such solutions could be relevant for other countries as well, and future research should therefore examine these possibilities.

### Strengths and limitations

This qualitative study has several strengths. Firstly, we included different methods (results from a systematic review, observations, and interviews) as well as different stakeholders (health professionals at hospitals, employees in municipalities, and participants in patient education), which is recommended within realist evaluation [39]. As a mechanism is an underlying construct [39], it is possible that the mechanisms are not discovered by the informants and revealed during the interviews, and therefore it is of special importance that we also conducted observation to get experience with the programs. Moreover, it is of importance that we applied theories to understand and interpret our findings. Secondly, we developed the program theory in an iterative process, which allowed an ongoing and careful refinement. Finally, we chose the informants for interviews and the locations for observation, so that these were widely represented with respect to geography, the size of the municipality, the participants, the content, and the referral to patient education.

The study also has some limitations. Among the participants from patient education, we only included those who had completed all or almost all sessions. Therefore, we did not include informants who had dropped out of the program, and thus we attained information from only those who presumably were satisfied with the program. Moreover, the participants chose to participate in group education, whereas others might refuse this offer because they are not interested in or able to be in a group. Consequently, we also attained information from only those who find it beneficial to be in a group. Another limitation concerns the size of the focus groups, as there were five participants in most groups. Some of the participants had little to say, perhaps because the group was too big to discuss sensitive subjects with, and therefore an individual interview could have benefitted them. Finally, the majority of the participants had just finished the program, and therefore we do not have a long follow-up period, though it could have provided us with more examples of the long-term outcomes. On the other hand, it is more likely that the participants better remember their experiences with the program when the follow-up period is short, and therefore this limitation is also a strength.

## Conclusion

No previous studies have developed a program theory of osteoporosis patient education. In this qualitative study, we developed a program theory illustrating the elements in osteoporosis patient education that provides an explanation of the potential effectiveness of such programs, which will we evaluated in a future study. We examined the mechanisms of change, including which activities should be implemented and which contextual factors should be considered to achieve potential outcomes. The findings show that activities, such as dissemination of information, exchange of experience, physical exercises, ergonomic movements, a combination of theory and practice, repetitions, and homework in osteoporosis patient education evoke motivation, recognizability, reassurance, and peer reflection. Additionally, we show that the following contextual factors activated the mechanisms: factors related to group education including the size and the heterogeneity of the group, the course of the disease, and training communities. This is of special importance for future implementation and evaluation of osteoporosis patient education.

### Supplementary Information

**Additional file 1. **Description of elements in program theory with examples of empirical data. 

## Data Availability

The data analyzed during the current study are not publicly available before the termination of a subsequent study but are available from the corresponding author on reasonable request.

## References

[1] Hernlund E, Svedbom A, Ivergård M, Compston J, Cooper C, Stenmark J (2013). Osteoporosis in the European Union: medical management, epidemiology and economic burden. A report prepared in collaboration with the International Osteoporosis Foundation (IOF) and the European Federation of Pharmaceutical Industry Associations (EFPIA). Arch Osteoporos.

[2] Odén A, McCloskey EV, Kanis JA, Harvey NC, Johansson H (2015). Burden of high fracture probability worldwide: secular increases 2010–2040. Osteoporos Int.

[3] Lips P, van Schoor NM (2005). Quality of life in patients with osteoporosis. Osteoporos Int.

[4] Guillemin F, Martinez L, Calvert M, Cooper C, Ganiats T, Gitlin M (2013). Fear of falling, fracture history, and comorbidities are associated with health-related quality of life among European and US women with osteoporosis in a large international study. Osteoporos Int.

[5] Ross PD, Davis JW, Epstein RS, Wasnich RD (1994). Pain and disability associated with new vertebral fractures and other spinal conditions. J Clin Epidemiol.

[6] Leidig G, Minne HW, Sauer P, Wüster C, Wüster J, Lojen M (1990). A study of complaints and their relation to vertebral destruction in patients with osteoporosis. Bone Miner.

[7] Papaioannou A, Wiktorowicz M, Adachi JD, Goeree R, Papadimitropoulos E, Bedard M (2000). Mortality, independence in living, and re-fracture, one year following hip fracture in Canada. J Soc Obstet Gynaecol Can.

[8] Eastell R, O'Neill TW, Hofbauer LC, Langdahl B, Reid IR, Gold DT (2016). Postmenopausal osteoporosis. Nat Rev Dis Primers.

[9] Ebeling PR, Daly RM, Kerr DA, Kimlin MG (2013). Building healthy bones throughout life: an evidence-informed strategy to prevent osteoporosis in Australia. Med J Aust.

[10] World Health Organization (2003). Prevention and management of osteoporosis: report of a WHO scientific group.

[11] Gregson CL, Armstrong DJ, Bowden J, Cooper C, Edwards J, Gittoes NJL (2022). UK clinical guideline for the prevention and treatment of osteoporosis. Arch Osteoporos.

[12] Lorentzon M, Johansson H, Harvey NC, Liu E, Vandenput L, McCloskey EV (2022). Osteoporosis and fractures in women: the burden of disease. Climacteric.

[13] Rothmann MJ, Jakobsen PR, Jensen CM, Hermann AP, Smith AC, Clemensen J (2018). Experiences of being diagnosed with osteoporosis: a meta-synthesis. Arch Osteoporos.

[14] Nielsen DS, Brixen K, Huniche L (2011). Men's experiences of living with osteoporosis: focus group interviews. Am J Mens Health.

[15] Nielsen D, Ryg J, Nielsen W, Knold B, Nissen N, Brixen K (2010). Patient education in groups increases knowledge of osteoporosis and adherence to treatment: a two-year randomized controlled trial. Patient Educ Couns.

[16] de Sire A, Invernizzi M, Baricich A, Lippi L, Ammendolia A, Grassi FA (2021). Optimization of transdisciplinary management of elderly with femur proximal extremity fracture: a patient-tailored plan from orthopaedics to rehabilitation. World J Orthop.

[17] Laird C, Benson H, Williams KA. Pharmacist interventions in osteoporosis management: a systematic review. Osteoporos Int. 2022. 10.1007/s00198-022-06561-1 PMC9852145 36239755

[18] Cornelissen D, de Kunder S, Si L, Reginster JY, Evers S, Boonen A (2020). Interventions to improve adherence to anti-osteoporosis medications: an updated systematic review. Osteoporos Int.

[19] Jensen A, Lomborg K, Wind G, Langdahl B (2014). Effectiveness and characteristics of multifaceted osteoporosis group education -a systematic review. Osteoporos Int.

[20] Morfeld J-C, Vennedey V, Müller D, Pieper D, Stock S (2017). Patient education in osteoporosis prevention: a systematic review focusing on methodological quality of randomised controlled trials. Osteoporos Int.

[21] Rubæk M, Hitz MF, Holmberg T, Schønwandt BMT, Andersen S (2022). Effectiveness of patient education for patients with osteoporosis: a systematic review. Osteoporos Int.

[22] Billington EO, Feasel AL, Kline GA (2020). At odds about the odds: women's choices to accept osteoporosis medications do not closely agree with physician-set treatment thresholds. J Gen Intern Med.

[23] Gold DT, Stegmaier K, Bales CW, Lyles KW, Westlund RE, Drezner MK (1993). Psychosocial functioning and osteoporosis in late life: results of a multidisciplinary intervention. J Womens Health.

[24] Grahn Kronhed A-C, Enthoven P, Spångeus A, Willerton C (2020). Mindfulness and modified medical yoga as intervention in older women with osteoporotic vertebral fracture. J Altern Complement Med.

[25] Kessenich CR, Guyatt GH, Patton CL, Griffith LE, Hamlin A, Rosen CJ (2000). Support group intervention for women with osteoporosis. Rehabil Nurs.

[26] Smulders E, Weerdesteyn V, Groen BE, Duysens J, Eijsbouts A, Laan R (2010). Efficacy of a short multidisciplinary falls prevention program for elderly persons with osteoporosis and a fall history: a randomized controlled trial. Arch Phys Med Rehabil.

[27] Tüzün S, Akyuz G, Eskiyurt N, Memis A, Kuran B, Icagasioglu A (2013). Impact of the training on the compliance and persistence of weekly bisphosphonate treatment in postmenopausal osteoporosis: a randomized controlled study. Int J Med Sci.

[28] Alp A, Kanat E, Yurtkuran M (2007). Efficacy of a self-management program for osteoporotic subjects. Am J Phys Med Rehabil.

[29] Bianchi ML, Duca P, Vai S, Guglielmi G, Viti R, Battista C (2015). Improving adherence to and persistence with oral therapy of osteoporosis. Osteoporos Int.

[30] Bergland A, Thorsen H, Kåresen R (2011). Effect of exercise on mobility, balance, and health-related quality of life in osteoporotic women with a history of vertebral fracture: a randomized, controlled trial. Osteoporos Int.

[31] Gold DT, Shipp KM, Pieper CF, Duncan PW, Martinez S, Lyles KW (2004). Group treatment improves trunk strength and psychological status in older women with vertebral fractures: results of a randomized, clinical trial. J Am Geriatr Soc.

[32] Skivington K, Matthews L, Simpson SA, Craig P, Baird J, Blazeby JM (2021). A new framework for developing and evaluating complex interventions: update of Medical Research Council guidance. BMJ.

[33] Craig P, Dieppe P, Macintyre S, Michie S, Nazareth I, Petticrew M (2008). Developing and evaluating complex interventions: the new Medical Research Council guidance. BMJ.

[34] Skivington K, Matthews L, Simpson SA, Craig P, Baird J, Blazeby JM (2021). Framework for the development and evaluation of complex interventions: gap analysis, workshop and consultation-informed update. Health Technol Assess.

[35] Cooper H, Booth K, Fear S, Gill G (2001). Chronic disease patient education: lessons from meta-analyses. Patient Educ Couns.

[36] Maidment I, Lawson S, Wong G, Booth A, Watson A, Zaman H (2020). Towards an understanding of the burdens of medication management affecting older people: the MEMORABLE realist synthesis. BMC Geriatr.

[37] Fletcher A, Jamal F, Moore G, Evans RE, Murphy S, Bonell C (2016). Realist complex intervention science: Applying realist principles across all phases of the Medical Research Council framework for developing and evaluating complex interventions. Evaluation (Lond).

[38] Patton DE, Cadogan CA, Ryan C, Francis JJ, Gormley GJ, Passmore P (2018). Improving adherence to multiple medications in older people in primary care: Selecting intervention components to address patient-reported barriers and facilitators. Health Expect.

[39] Pawson R, Tilley N (1997). Realistic evaluation.

[40] Bonell C, Fletcher A, Morton M, Lorenc T, Moore L (2012). Realist randomised controlled trials: a new approach to evaluating complex public health interventions. Soc Sci Med.

[41] Pawson R (2013). The science of evaluation: a realist manifesto.

[42] Gertler PJ, Martinez S, Premand P, Rawlings LB, Vermeersch CMJ (2016). Impact evaluation in practice.

[43] Dalkin SM, Greenhalgh J, Jones D, Cunningham B, Lhussier M (2015). What's in a mechanism? Development of a key concept in realist evaluation. Implement Sci.

[44] Pawson R, Tilley N. Realistic evaluation. Thousand Oaks, Calif.: SAGE Publications Ltd; 1997.

[45] Videnscenter for Knoglesundhed. Osteoporoseskoler i Danmark: en afdækning af uddannelses- og rehabiliteringstilbud. 2019.

[46] Sundhed.dk. Sundhedstilbud [Available from: https://www.sundhed.dk/borger/guides/sundhedstilbud/ Accessed 3 Aug 2022.

[47] Osteoporoseforeningen. Find en osteoporoseskole [Available from: https://www.osteoporose-f.dk/stoette-og-hjaelp/osteoporoseskole-forloeb/ Accessed 3 Aug 2022

[48] Funnell S, Rogers P. Purposeful program theory: effective use of theories of change and logic models. 1 ed. United States of America: John Wiley And Sons Ltd; 2011.

[49] Hammersley M, Atkinson P (2007). Ethnography: principles in practice.

[50] Jamal F, Fletcher A, Shackleton N, Elbourne D, Viner R, Bonell C (2015). The three stages of building and testing mid-level theories in a realist RCT: a theoretical and methodological case-example. Trials.

[51] Mason J (2006). Qualitative researching.

[52] Braun V, Clarke V (2006). Using thematic analysis in psychology. Qual Res in Psychol.

[53] Fisher JD, Fisher WA (1992). Changing AIDS-risk behavior. Psychol Bull.

[54] Fisher WA, Fisher JD, Harman J. The Information–Motivation–Behavioral Skills Model: a general social psychological approach to understanding and promoting health behavior. In: Suls J, Wallston KA, editors. Social psychological foundations of health and illness: Blackwell Publishing Ltd; 2003. p. 82–106.

[55] Tanaka S, Stenner P, Cromby J, Motzkau J, Yen J, Haosheng Y (2011). The notion of embodied knowledge. Theoretical psychology: global transformations and challenges.

[56] Witte K (1992). Putting the fear back into fear appeals: the extended parallel process model. Commun Monogr.

[57] Perloff RM (2003). The dynamics of persuasion: communication and attitudes in the 21st century.

[58] Mead S, Hilton D, Curtis L (2001). Peer support: a theoretical perspective. Psychiatr Rehabil J.

[59] Jensen A, Lomborg K, Langdahl B, Wind G, Jensen AL, Langdahl BL (2016). Managing a bone healthy lifestyle after attending multifaceted group education. Calcif Tissue Int.

[60] Weston JM, Norris EV, Clark EM (2011). The invisible disease: making sense of an osteoporosis diagnosis in older age. Qual Health Res.

[61] Nielsen D, Huniche L, Brixen K, Sahota O, Masud T (2013). Handling knowledge on osteoporosis - a qualitative study. Scand J Caring Sci.

[62] Bennell KL, Matthews B, Greig A, Briggs A, Kelly A, Sherburn M (2010). Effects of an exercise and manual therapy program on physical impairments, function and quality-of-life in people with osteoporotic vertebral fracture: a randomised, single-blind controlled pilot trial. BMC Musculoskelet Disord.

[63] Papaioannou A, Adachi JD, Winegard K, Ferko N, Parkinson W, Cook RJ (2003). Efficacy of home-based exercise for improving quality of life among elderly women with symptomatic osteoporosis-related vertebral fractures. Osteoporos Int.

[64] Province MA, Hadley EC, Hornbrook MC, Lipsitz LA, Miller JP, Mulrow CD (1995). The effects of exercise on falls in elderly patients. A preplanned meta-analysis of the FICSIT Trials. Frailty and Injuries: Cooperative Studies of Intervention Techniques. Jama.

[65] Brooke-Wavell K, Skelton DA, Barker KL, Clark EM, De Biase S, Arnold S, et al. Strong, steady and straight: UK consensus statement on physical activity and exercise for osteoporosis. Br J Sports Med. 2022: 10.1136/bjsports-2021-104634. 10.1136/bjsports-2021-104634 PMC9304091 35577538

[66] Zangi HA, Ndosi M, Adams J, Andersen L, Bode C, Boström C (2015). EULAR recommendations for patient education for people with inflammatory arthritis. Ann Rheum Dis.

[67] Holmberg T, Möller S, Rothmann MJ, Gram J, Herman AP, Brixen K (2019). Socioeconomic status and risk of osteoporotic fractures and the use of DXA scans: data from the Danish population-based ROSE study. Osteoporos Int.

[68] Kutsal YG, Atalay A, Arslan S, Başaran A, Cantürk F, Cindaş A (2005). Awareness of osteoporotic patients. Osteoporos Int.

