# Supplementary material for: Development of a program theory for osteoporosis patient education in Denmark: a qualitative study based on realist evaluation
Source: BMC Geriatr. 2024 Apr 16;24:346. doi: 10.1186/s12877-024-04957-8 (PMC11022455; doi:10.1186/s12877-024-04957-8)
Supplement: Supplementary file 1 — Additional file 1. Description of elements in program theory with examples of empirical data. [file 12877_2024_4957_MOESM1_ESM.docx]

# Additional file 1: Description of elements in program theory with examples of empirical data

| **Short-term outcomes** | **Description** | **Examples of empirical data** |
| --- | --- | --- |
| More knowledge | The participants get more knowledge when participating in patient education. This knowledge may include different topics, but typically it is knowledge about osteoporosis, diet, medication, side effects, pain, and assistive technology. | Focus group interview with participants:  Par14: [...] But I think I also got a lot out of the information about the different types of medication. Because what happens if you start taking something and are, could there be some side effects? Should you then have something else? Can you not take that either and? |
| More physical activity | As result of the patient education, the participants become more physically active. This may be all kinds of exercises, but especially weight-bearing exercises. | Focus group interview with participants:  Par3: well, in this program at least, I’ve learned about the body’s movements. And what you can and should do. And what you can’t and shouldn’t do. And it’s also given me encouragement to do the exercises. Well, I haven’t been the best at doing exercises before, but this has really encouraged me to do them. And the same goes for all that with walking. I mean, walking, I’ve probably done it kind of on and off, but not to the extent that I’ve now been told that you should. |
| Healthier diet | The participants become more aware of their diet and make sure it is rich in, e.g., calcium and aligned with their supplements. | Interview with health professional at hospital:  Hea3: [...] It can be the diet, they get an understanding of which foods contain calcium, and what should I actually eat for it to be bone friendly. So this feeling of control, where they themselves want, can help a little with managing and making a difference, I think, is very important. |
| Better ergonomic habits | During the sessions, especially with the occupational therapist, the participants learn how to move ergonomically, e.g., regarding cleaning, cooking, gardening, etc. | Focus group interview with participants:  Par5: but you pay attention. It’s like, it was drummed in, kind of. Like saying, oh, there are all those things with the laundry basket. Or all that with vacuuming. That’s just one of the very worst. So, I got hold of a vacuum cleaner. I can use it with one hand in my pocket, one like that. |
| More sense of control in the health sector | The participants get advice on when to contact their GP and what they could say during the consultation. | Focus group interview with participants:  Par5: [...] And then I asked to get those DXA scans [measuring bone density] myself, because I thought it was important. Also because it was very important for me to know what my score [reflecting the severity of osteoporosis] was. And I remember that, [name of teacher] drew a lot of attention to that. She said, you can, you can just ask your GP about it, just like getting a blood test for vitamin D. Because they don’t like that very much. |
| **Medium-term outcomes** | **Description** | **Examples of empirical data** |
| More self-efficacy | As a result of participation in patient education and as a result of the short-term outcomes, e.g., more knowledge and physical activity, then the participants trust that they can handle their disease, i.e., they attain more self-efficacy. | Focus group interview with participants:  Par17: [...] And then suddenly, then you think, that you know something about it. I found that reassuring because now you can take care of it yourself, I think. I’m not sure, it might be that I’m wrong, but I’m doing my best at least. That, I think, that’s a nice thing to know. I do what I can do, what I can do myself, and then I can’t do any more than that. |
| Better physical function | The participants may achieve a better physical function because of some of the short-term outcomes, e.g., more physical activity. Physical function may include more strength, mobility, and fitness. | Interview with employee in municipality:  Emp2: yes, well, I would say, when they’re finished with us, they should feel that they have physically improved a bit. At least that’s what they’re saying to us.  The results from the systematic review (1) |
| Better balance | Because of the exercise sessions in patient education and because of some of the short-term outcomes, e.g., more physical activity, the participants achieve better balance | Interview with employee in municipality:  Emp2: well, yes, we practice balance every single time. I put it into words as well, we have it in the teaching too. I mean, the thing about being over 60, your balance decreases if you don’t actively do something about it. So, we spend quite a lot of energy on it in the warm-up, and I also encourage them to do daily balance challenges, when they’re waiting for the water to boil, and when they’re brushing their teeth and things like that. So, we actually put a lot of energy into it.  The results from the systematic review (1) |
| Fewer falls | The participants may experience fewer falls as a result of patient education and some of the short-term outcomes, e.g., more knowledge and more physical activity. | Interview with employee in municipality:  Emp3: we, but we talk about preventing falls, and we talk about, have a topic called balance, where we cover and talk about fall prevention and also what they should be aware of in terms of preventing falls. […] And when we talk about falls, well, then the physical part of it, well, what is it going to take, that we have good balance, we prevent these falls. Strength in the legs is important. And then fall prevention in general, to do with, well, do their glasses fit their eyes, is the strength right, are loose wires lying around, rugs and all these things at home, what shoes do they wear, what surface do they walk on, be aware of that.  The results from the systematic review (1) |
| More adherence | The participants may become more adherent regarding their osteoporosis medication. During the patient education program, they are encouraged to contact their GP if they experience side effects. Therefore, they may actually contact their GP and continue taking their medication or new medication. | Interview with employee in municipality:  Emp4: […] I know there are also some citizens who tend to, if they can’t tolerate it or have side effects, then they stop taking it without involving their doctor. So we have a good talk about why you should involve your doctor. And I do understand that when you experience significant side effects, then you think, I’ll stop taking this right away. But then they don’t follow up with their own doctor. |
| Better pain management | Not all participants in osteoporosis patient education experience pain, but despite that, all participants get tools to manage pain if or when they experience it in the future. | Focus group interview with participants:  Par9: well, I’ve learned some exercises that I can use, even when I come home to my own place. So that it helps, that my back doesn’t hurt as much. |
| Better psychological well-being | The participants may experience better psychological well-being, which includes psychological symptoms, stress, and welfare. | Interview with employee in municipality:  Emp4: […] So I also want to say that many of those who come in are elderly, and elderly people who are co-morbid, and some of them, we can see, also have a tendency, you know, tend to be lonely. Depression. Stress. […] But these are also some of the things the citizens experience, in many ways, a little bit, without being able to estimate how many, but experience improvement by this when being exposed a little bit in these communities. Getting out, breaking out of all that. So, mental health is absolutely part of what, we absolutely also do everything possible to move the citizen within the parameters it’s possible, and the citizens themselves are willing to.  The results from the systematic review (1) |
| **Long-term outcomes** | **Description** | **Examples of empirical data** |
| Better quality of life | As a result of the short-term and medium-term outcomes, e.g., better psychological wellbeing, the participants may achieve better quality of life. | Interview with health professional at hospital:  In: [...] If you had to look at this, kind of, in relation to the overall purposes, what do you think could be the absolute ultimate effects of attending osteoporosis patient education? [...]  Hea2: Yes, well, the ultimate goal for us is to increase the quality of life for the individual, you could say. |
| Fewer fractures | The participants may experience fewer fractures in the longer term. Fractures include both vertebral and non-vertebral fractures. | Interview with employee in municipality:  In: what do you think is the purpose for the osteoporosis patient education? […]  Emp3: the long-term aim is hopefully to prevent fractures. You could say that the citizens who come here to the group, they’ve already had this diagnosis, so it’s not to prevent them getting osteoporosis, but in the long term to prevent fractures. |
| Better daily functional capacity | Daily functional capacity includes tasks carried out on a daily basis, such as eating, dressing, cooking, shopping, cleaning, etc. | Interview with employee in municipality:  Emp6: well, our thoughts and ideas are, of course, about the big picture, that we would like them to be as self-reliant for as long as possible. And take responsibility for living as good a life as possible with osteoporosis, right? |
| **Contextual factors** | **Description** | **Examples of empirical data** |
| Course of the disease | The course or progression of the disease includes the extent to which the individual has experienced, e.g., fractures, pain, or functional impairments as a result of their disease. | Interview with employee in municipality:  Emp6: [...] But our experience is also that it’s, that it’s the newly diagnosed and those who have maybe had their first vertebral fracture or just got their diagnosis, they are the ones we can help the most. [...] Because that’s where we can influence them the most in terms of how they can make some changes. |
| Training community | Training community covers family, friends, or citizens whom the participants do physical exercises with. | Interview with health professional at hospital:  Hea1: [...] Then they had, they found someone to train with, so they were to a much greater degree, they stuck with it. Well. If you find someone you can walk with, someone you can go to the gym with, someone you can, well, go down to the health center with. |
| Group education | In these particular programs, the participants meet in groups of 6-12 participants. | Focus group interview with participants:  Par11: [...] But I also think that the thing with exercising, that you just like... well, like today, when we were in there [in the gym], and I was lying on the ball, then I could feel, I just had to pee.  [Several laughs]  Par11: but there wasn’t anything to do, I just had to get it over with, you know. But then we also encouraged each other to say, now we’ll make a little more effort, right? After all, I could easily have stood up and then left, and then, oh well. |
| Heterogeneity of the group | The participants in the groups are very different with regard to age, physical function, pain, co-morbidity, and time since diagnosis. | Interview with employee in municipality:  Emp6: [...] But we have both, and it’s also a challenge for us that we can have Inga who’s 60, who is physically active, still working, and then we can have Egon at 82 who has, well, a hip fracture and four vertebral fractures and other things too, which complicates that picture.  Observation:  During observation it was seen how some participants are different regarding, e.g., age and functional abilities. |
| Transport | Transportation to the sessions may be of importance, e.g., if the participant has a long distance to travel. | Interview with employee in municipality:  Emp3: [...]But it’s very few people who say it’s a problem, but we’ve experienced a few who... we had someone in the last group who simply had to stop the program early because it was too difficult to take the bus out here. |
| **Inputs** | **Description** | **Examples of empirical data** |
| Staff  Interdisciplinarity  Pedagogic tools  Classrooms  Training facilities  Kitchen for training | Input covers the means required for conducting osteoporosis patient education. Staff is required, and the different professions should be able to cooperate (interdisciplinarity). Moreover, they should have pedagogic tools to be able to teach. Classrooms for dissemination of information and training facilities for physical exercises are required. A kitchen for training of ergonomic habits may be beneficial. | Interview with employee in municipality:  Emp5: yes, well, you know, we learn differently, so some have to hear it, some have to see it, and others have to do it. So, we’re trying to include all the components in relation to that, right? And then they can bring their own experiences into it as well, right?  Observation:  During observation it was seen how different staff held the sessions. In one municipality it was seen how two physiotherapists and one nurse held a session with strength training and aerobic exercises, and afterwards they met to talk about the session and ensure interdisciplinarity.  Descriptions of Danish osteoporosis patient educations programs (2)  Overview of municipalities conducting osteoporosis patient education (3) |
| **Activities** | **Description** | **Examples of empirical data** |
| Dissemination of information  Exchange of experience  Physical exercises  Ergonomic movements  Combination of theory and practice  Repetitions  Homework  Differentiation | Activities covers the content of osteoporosis patient education. During the sessions, the participants should receive information about osteoporosis, diet, medication, etc., and they should have the opportunity to exchange experiences. Moreover, they should participate in physical exercises and do ergonomic movements. They should have a combination of theory (e.g., information about ergonomics) and practice (e.g., try to make the bed). The participants should get the same information and do the same exercises several times (repetitions), and they should do homework between each session. Finally, the teachers should differentiate the physical exercises so that they are adjusted to the participants’ functional capacity. | Interview with employee in municipality:  Emp5: […] So we get into, well, what is it you, I mean, what is osteoporosis and how does it come about, and how many people get it? […] And what lies, what are the recommendations about it? And, well, about diet, but also about ergonomics, and about physical exercises.  Observation:  During observation, most of the activities were seen. For instance, in one municipality we observed the participants making the bed, ironing clothes, and doing the laundry while taking care of their back.  Descriptions of Danish osteoporosis patient educations programs (2)  Overview of municipalities conducting osteoporosis patient education (3) |
| **Outputs** | **Description** | **Examples of empirical data** |
| Program conducted  Participants attended the whole program | To achieve the intended outcomes, the program should be conducted, and the participants should attend the sessions. In these cases, the programs are conducted during 6-12 weeks with attendance 1-2 times a week. | Interview with employee in municipality:  Emp1: […] But I would say that, you know, we’ve been together 16 times […] So that’s a long time to be able to carry out a process, instead of that you just get told things. I mean, if all of this, if it was boiled down to just two theme days, for example, I don’t think you would get the same out of it, because people, they just need to go home and work with it.  Descriptions of Danish osteoporosis patient educations programs (2)  Overview of municipalities conducting osteoporosis patient education (3) |

*DXA* Dual-energy X-ray Absorptiometry

References

1. Rubæk M, Hitz MF, Holmberg T, Schønwandt BMT, Andersen S. Effectiveness of patient education for patients with osteoporosis: a systematic review. Osteoporos Int. 2022;33(5):959-77.

2. Sundhed.dk. Sundhedstilbud [Available from: <https://www.sundhed.dk/borger/guides/sundhedstilbud/> Accessed 3 August 2022.

3. Videnscenter for Knoglesundhed. Osteoporoseskoler i Danmark: en afdækning af uddannelses- og rehabiliteringstilbud. 2019.
